# Targeting TIGIT Inhibits Bladder Cancer Metastasis Through Suppressing IL-32

**DOI:** 10.3389/fphar.2021.801493

**Published:** 2022-01-05

**Authors:** Kang Wu, Jun Zeng, Xulian Shi, Jiajia Xie, Yuqing Li, Haoxiang Zheng, Guoyu Peng, Guanghui Zhu, Dongdong Tang, Song Wu

**Affiliations:** ^1^ Department of Urology, The Third Affiliated Hospital of Shenzhen University (Luohu Hospital Group), Shenzhen, China; ^2^ Key Laboratory of Regenerative Biology, Guangdong Provincial Key Laboratory of Stem Cell and Regenerative Medicine, South China Institute for Stem Cell Biology and Regenerative Medicine, Guangzhou Institutes of Biomedicine and Health, Chinese Academy of Sciences, Guangzhou, China; ^3^ Shenzhen Following Precision Medicine Research Institute, Shenzhen, China; ^4^ Department of Genetics and Cell Biology, College of Life Sciences, Chongqing Normal University, Chongqing, China; ^5^ Medical Laboratory of Shenzhen Luohu People’s Hospital, Shenzhen, China; ^6^ Teaching Center of Shenzhen Luohu Hospital, Shantou University Medical College, Shantou, China; ^7^ Department of Urology and Guangdong Key Laboratory of Urology, The First Affiliated Hospital of Guangzhou Medical University, Guangzhou, China

**Keywords:** bladder cancer, TIGIT, IL-32, metastasis, immunotherapy

## Abstract

Bladder cancer is a highly metastatic tumor and one of the most common malignancies originating in the urinary tract. Despite the efficacy of immune checkpoints, including programmed cell death-1 (PD-1) and cytotoxic T-lymphocyte-associated protein 4 (CTLA-4), the effect of immunotherapy for bladder cancer remains unsatisfactory. Therefore, it is urgent to develop new targets to expand immunotherapeutic options. In this study, we utilized single-cell sequencing to explore the cell composition of tumors and detected a subset of Treg cells with high expression of T-cell immunoreceptor with immunoglobulin and immunoreceptor tyrosine-based inhibitory motif domain (TIGIT) and interleukin (IL)-32. The antitumor immune response was suppressed by this subset of Treg cells, while IL-32 promoted bladder cancer metastasis. Nevertheless, targeting TIGIT not only reversed immunosuppression by restoring the antitumor immune response mediated by T cells but also suppressed the secretion of IL-32 and inhibited the metastasis of bladder cancer cells. Thus, our study provided novel insights into immunosuppression in bladder cancer and highlighted TIGIT as a novel target for immunotherapy of bladder cancer. We also illustrated the mechanism of the dual effect of targeting TIGIT and revealed the metastasis-promoting effect of IL-32 in bladder cancer. Collectively, these findings raise the possibility of utilizing TIGIT as a target against bladder cancer from the bench to the bedside.

## Introduction

Treatment strategies for patients with solid tumors have traditionally been based on three different options: surgery, targeted therapies, and cytotoxic therapy (chemotherapy or radiation therapy) (Chauvin and Zarour, 2020). Immunotherapy has only recently emerged as a novel therapeutic paradigm in our armamentarium (Solomon and Garrido-Laguna, 2018). Unfortunately, the vast majority of patients cannot benefit from immunotherapy (Ma et al., 2021). Thus, it is necessary to explore new targets for immunotherapy to facilitate the improved treatment of solid tumors.

T-cell immunoreceptor with immunoglobulin and ITIM domain (TIGIT, also called WUCAM, Vstm3, or VSIG9) is a receptor of the Ig superfamily, which plays a critical role in the regulation of immunoresponses (Chauvin and Zarour, 2020). In particular, TIGIT is an immunoreceptor inhibitor checkpoint that has been implicated in tumor immunosurveillance (Manieri et al., 2017; Harjunpaa and Guillerey, 2020). Preclinical models, such as colorectal cancer and melanoma models, have suggested the synergy of anti-TIGIT antibodies with anti-PD-1/PD-L1 antibodies (Curran, 2018). Interestingly, TIGIT molecules have been identified on the surface of CD8^+^ T cells in bladder cancer, but their function has not been well characterized (Han et al., 2021).

Interleukin-32 (IL-32) is a novel cytokine regulating cancer development and inflammation (Kwon et al., 2018). IL-32 is initially expressed selectively in mitogen-activated T cells and NK cells, and its expression is strongly augmented by microbes, mitogens, and other cytokines (Kim, 2014; Lee et al., 2019). Despite IL-32 being induced mainly by pathogens and proinflammatory cytokines, its expression is more prominent in immune cells than in nonimmune tissues (Khawar et al., 2016). Moreover, IL-32 is expressed in various human tissues and organs such as the spleen, thymus, leukocytes, lung, small intestine, colon, prostate, heart, placenta, liver, muscle, kidney, pancreas, and brain (Oh et al., 2011; Yun et al., 2018; Baselli et al., 2020). Previous studies have demonstrated that IL-32 regulates cell proliferation, metabolism, and immune response and is also involved in the pathological regulation or protection against inflammatory diseases (Netea et al., 2008; Kwon et al., 2018; Palstra et al., 2018). IL-32 has also been involved in various cancers, including renal cancer, esophageal cancer, lung cancer, gastric cancer, breast cancer, pancreatic cancer, lymphoma, osteosarcoma, breast cancer, colon cancer, and thyroid carcinoma (Tsai et al., 2014; Hong et al., 2017; Wen et al., 2019). However, other studies have suggested that IL-32 decreases tumor development, including cervical cancer, colon cancer, prostate cancer, melanoma, pancreatic cancer, liver cancer, and chronic myeloid leukemia (Nishida et al., 2009; Lee et al., 2011; Park et al., 2012; Sloot et al., 2018). Notably, the expression of IL-32 receptor (IL-32R) on the surface of epithelial cells is mediated by the stimulation of interferon gamma (IFN-γ), as previously reported (Aass et al., 2021). Although a higher expression of IL-32 has been demonstrated in bladder cancer, its function has not been well characterized (Yang et al., 2020).

Taken together, our study demonstrated the enrichment of TIGIT^+^ Treg cells in bladder cancer tissues. Furthermore, Treg cells expressed IL-32 to promote the migration and invasion of bladder cancer cells. In addition, targeting TIGIT with anti-TIGIT monoclonal antibodies suppressed the metastasis of bladder cancer and reversed its antitumor activities. Thus, we have provided novel insights into the function of IL-32 in bladder cancer and the effect of anti-TIGIT monoclonal antibodies against bladder cancer.

## Materials and Methods

### Single-Cell RNA Sequencing

The BD Rhapsody system was used to capture the transcriptomic information of bladder-derived single cells with BD Rhapsody WTA Amplification Kit (Becton, Dickinson and Company, BD Biosciences). Single-cell capture was achieved by random distribution of a single-cell suspension across >200,000 microwells using a limited dilution approach. Beads with oligonucleotide barcodes were added to saturation, so that a single bead was paired with a single cell in a microwell. Cell lysis buffer was added so that the polyadenylated RNA molecules hybridized to beads. Beads were collected in a single tube for reverse transcription. Upon complementary DNA (cDNA) synthesis, each cDNA molecule was tagged on the 5 end, that is, the 3 end of the messenger RNA (mRNA) transcript, with a unique molecular identifier (UMI) and cell label indicating its cell of origin. Whole transcriptome libraries were prepared using the BD Rhapsody single-cell whole-transcriptome amplification workflow. In brief, second-strand cDNA was synthesized, followed by ligation of the WTA adaptor for universal amplification. Eighteen PCR cycles were used to amplify adaptor-ligated cDNA products. Sequencing libraries were prepared using random priming PCR of whole-transcriptome amplification products to enrich the 3 end of transcripts linked with the cell label and UMI. Sequencing libraries were quantified using a High-Sensitivity DNA chip (Agilent) on a Bioanalyzer 2200 and the Qubit High-Sensitivity DNA assay (Thermo Fisher Scientific). The library for each sample was sequenced using an Illumina sequencer (Illumina) on a 150-bp paired-end run.

### Single-Cell RNA Statistical Analysis

scRNA-seq data analysis was performed by NovelBio Co., Ltd. using the NovelBrain cloud analysis platform. We applied fast p using default parameter settings that filtered the adaptor sequence and removed low-quality reads to achieve clean data (Chen et al., 2018). UMI tools were applied for single-cell transcriptome analysis to identify the cell barcode whitelist (Smith et al., 2017). UMI-based clean data were mapped to the human genome (Ensemble version 91) utilizing STAR mapping with customized parameters from the UMI tools standard pipeline to obtain the UMI counts of each sample (Dobin et al., 2013). Cells containing over 200 expressed genes and mitochondrial UMI rates below 20% passed the cell quality filtering, and mitochondrial genes were removed from the expression table. The Seurat package (version: 2.3.4, https://satijalab.org/seurat/) was used for cell normalization and regression based on the expression table according to the UMI counts of each sample and percentage of mitochondrial rate to obtain scaled data. Principal component analysis (PCA) was constructed based on the scaled data with the top 2,000 highly variable genes, whereas the top 10 principals were used for the construction of t-SNE and UMAP.

Utilizing the graph-based cluster method (resolution = 0.8), we acquired the results of unsupervised cell clusters based on the PCA top 10 principals and calculated the marker genes using the FindAllMarkers function with the Wilcoxon rank-sum test algorithm under the following criteria: (1) lnFC >0.25; 2. *p* value < 0.05; 3. min. pct >0.1. To identify the cell type in detail, clusters of the same cell type were selected for re-tSNE analysis, graph-based clustering, and marker analysis.

### Estimation of Copy Number Variations

Cells were defined as endothelial cells, fibroblast cells, macrophages, epithelial cells, T cells, and B cells and were used as a reference to identify somatic copy number variations (CNVs) with the R package infercnv (v0.8.2). We scored each cell according to the extent of the CNV signal, defined as the mean of squares of CNV values across the genome. Putative malignant cells were then defined as those with a CNV signal above 0.05 and CNV correlation above 0.5.

### Patient Specimens

Specimens were collected from 24 patients with bladder cancer at the Luohu Hospital. The protocol adopted in this study conformed to the ethical guidelines of the 1975 Helsinki Declaration and was approved by the Ethics Review Committee of Luohu Hospital. Clinical data, pathological features, AJCC staging, and other data were collected for follow-up visits and subsequent analyses. Tumor staging was estimated according to the histological classification criteria proposed by the International Union for Cancer. A total of 24 tumor and adjacent normal tissue pairs were collected from patients who underwent bladder cancer resection between 2018 and 2019. Clinical samples are listed in **Supplementary Table I**.

### Cell Culture

T24 and EJ human bladder cancer cell lines, and the MBT2 mouse bladder cancer cell line, were purchased from the American Type Culture Collection (ATCC). All cell lines were identified by short-tandem repeat analysis and were guaranteed to be used within 6 months. The most recent test was performed 3 months ago. All cell lines were maintained in Roswell Park Memorial Institute (RPMI) 1640 (Invitrogen) supplemented with 1 × 10^7^ U/L penicillin (Invitrogen), 10 mg/L streptomycin (Sigma-Aldrich), and 10% fetal bovine serum (FBS) (Gibco) in a humidified incubator at 37°C and 5% CO_2_ atmosphere, following cell culture guidelines.

### *In Vivo* Tumor Model

MBT2-luciferase cells were established as previously described. Briefly, the full-length cDNA sequence of the luciferase gene was synthesized (Shanghai Generay Biotech Co., Ltd.) and cloned into the pMSCV-puro retroviral vector plasmid (Clontech Laboratories Inc.) to generate the pMSCV-luciferase plasmid. pMSCV-luciferase was cotransfected with the pIK packaging plasmid into 293T cells using the calcium phosphate transfection method. Then, 48 h after transfection, the supernatants were collected and incubated with MBT2 cells. The infectious mixture was incubated for 24 h in the presence of polybrene (2.5 μg/ml). Puromycin (4 μg/ml) was then used to select stably transfected cells over a 12-day period. Female C57B6/J mice (6–8 weeks of age) were housed under specific pathogen-free (SPF) conditions. All animal care and experiments were conducted in accordance with the guidelines provided by the Animal Center of Luohu Hospital and were approved by the Experimental Animal Ethics Committee of Luohu Hospital. In the mouse lung metastasis model, 1 × 10^6^ MBT2-luciferase cells were intravenously injected into two groups of mice. Mice were euthanized at 4 and 6 weeks after injection to observe tumor cell metastasis in the lungs. Metastases were confirmed by hematoxylin and eosin (H&E) staining. The bladder cancer orthotropic mouse model was established by injecting 1 × 10^6^ MBT2-luciferase cells into the bladder wall of mice after anesthesia. After 18 days, each mouse was intravenously injected with 100 μg of neutralizing antibodies every 3 days. Mice were examined daily, and the date of death was recorded for each mouse. To establish the subcutaneous model, MBT2 cells (1 × 10^6^ in 50 μl FBS-free medium containing 20% Matrigel) were injected into the left flank of recipient mice. After 18 days, each mouse was intravenously injected with 100 μg of neutralizing antibodies every 3 days. Mice were examined daily, and the date of death was recorded for each mouse.

### *In Vivo* Bioluminescence

C57B6/J mice inoculated with MBT2-luciferase cells were anesthetized by intraperitoneal injection of sodium pentobarbital (50 mg/kg) and then injected with D-luciferin potassium salt (150 mg/kg). After 15 min, the bioluminescence of mice was tracked and imaged (AniView100, Biolight Biotechnology Co., Ltd.).

### Flow Cytometry

Cells were analyzed by flow cytometry, as previously described (Wu et al., 2019a). Single-cell suspensions were prepared from the bladder, spleen, blood, or tumors of individual mice. For cell staining, cells were preincubated in 0.1% bovine serum albumin (BSA)/phosphate-buffered saline (PBS) solution containing 10 μg/ml anti-FcgRII/III (2.4 G2) (BD Pharmingen) for 10 min at 4°C. Cells were then stained for 20 min at 4°C with primary antibodies. For intracellular cytokine staining, cells were stimulated with 100 ng/ml phorbol 12-myristate 13-acetate (PMA) (Sigma-Aldrich) and 1 μg/ml ionomycin (Sigma-Aldrich) in the presence of 5 μg/ml brefeldin A (Sigma-Aldrich) for 4 h. Cells were washed twice in PBS, fixed, and permeabilized using the BD Cytofix/Cytoperm™ fixation/permeabilization kit. Stained samples were analyzed using the BD FACSAria II system (BD Biosciences). Flow cytometric data were analyzed using the FlowJo software (Tree Star).

The primary antibodies used in the study included anti-FOXP3 (MF-14), anti-CD25 (3C7), anti-CD3 (145-2C11), anti-TIGIT (1G9), anti-CD8 (53-6.7), anti-IFN-γ (XMG1.2), and anti-CD4 (RM4-5).

### Mice

C57BL/6J mice were purchased from Vital River Laboratories (Beijing, China) and bred under specific pathogen-free (SPF) conditions at Luohu Hospital. All mouse experiments were approved by the Institutional Animal Care and Use Committee of Luohu Hospital.

### Scratch Wound Healing Assay

To evaluate their migration ability after treatment, T24 cells and EJ cells were seeded into six-well cell culture plates and grown in RPMI 1640 medium containing 10% FBS for 24 h to form an adherent monolayer with a degree of fusion approaching 70%–80%. A cross was scratched in the culture plate using a 200-μl sterile pipette tip, and detached cells were removed by gently washing the well twice with PBS. Fresh medium was added to the culture wells, and cells were cultured with IFN-γ (10 ng/ml) or IL-32 (10 ng/ml) for an additional 24 h. Transplanted cells were photographed (40×) using an inverted optical microscope (Zeiss) at 0 and 24 h to monitor the migration of cells to the wound area and calculate wound closure.

### Invasion Assays

To assess the invasion ability of T24 and EJ cells, matrix gel (Corning) was thawed overnight at 4°C and diluted in serum-free medium RPMI 1640 at a scale of 1:7. Next, a Transwell was coated in a 24-well plate with 50 μl diluted matrix gel and incubated at 37°C for 30 min. Then, 1 × 10^5^ cells in serum-free medium were seeded into the Transwell upper chamber. Concomitantly, 500 μl RPMI 1640 medium supplemented with 10% FBS was added to the lower Transwell chamber and incubated with IFN-γ (10 ng/ml) or IL-32 (10 ng/ml) at 37°C and 5% CO_2_ for 24 h. The Transwell chamber was removed, washed thrice with PBS, and dried using a cotton swab. Cells were fixed with 4% paraformaldehyde (Sigma-Aldrich) and stained with crystal violet for 5 min. The Transwell chamber was photographed, and cells were counted under an optical microscope (40×) (Zeiss).

### Immunohistochemistry

Tumor and animal tissues were fixed with paraformaldehyde, dehydrated, and cleared using a gradient of alcohol solutions and xylene, respectively. Subsequently, tissues were embedded in paraffin. Paraffin-embedded tissues were sliced continuously (3.5°C) using a HistoCore AUTOCUT system (Leica), mounted, dewaxed, and rehydrated. For antigen retrieval, sections (3–4 μm) were pretreated in a microwave oven with citric acid buffer (pH 6.0) for 12 min and then cooled to 25°C in deionized water. Sections were then incubated in methanol containing 3% hydrogen peroxide to inhibit endogenous peroxidase activity. After washing with PBS for 5 min, sections were incubated with normal goat serum at 37°C for 1 h, and incubated overnight with anti-IL-32 and anti-TIGIT (Proteintech) at 4°C. Sections were then washed again with PBS containing 0.1% BSA and incubated with rabbit anti-goat IgG and horseradish peroxidase (HRP)-linked antibodies (Proteintech). Specific binding was assessed using diaminobenzidine; hematoxylin and eosin staining (Solarbio) was performed as counterstaining. Paraffin sections were photographed using the imaging system of an ortho DM6 B microscope (Leica).

### Real-Time Quantitative PCR

Total RNA was extracted and purified using a standard procedure. Total purified RNA was reverse-transcribed into cDNA using a reverse transcription kit (k1622, Thermo Scientific, United States). Primers were designed using the Primer-BLAST tool (https://www.ncbi.nlm.nih.gov/tools/primer-blast/): forward primer, 5′-CGG​AAT​TCA​TGT​GCT​TCC​CGA​AGG​TCC-3′; reverse primer, 5′-CCG​CTC​GAG​TCA​TTT​TGA​GGA​TTG​GGG​TTC-3′. Quantitative real-time PCR (qRT-PCR) was performed on an ABI 7500 real-time fluorescent quantitative PCR instrument using SYBR Green (420A, Takara). Cycle threshold values of genes of interest were normalized to that of GAPDH: forward primer, 5′-CCC​AGC​TTA​GGT​TCA​TCA​GGT-3′; reverse primer, 5′-TAC​GGC​CAA​ATC​CGT​TCA​CA-3′.

### Immunofluorescence Staining

Immunofluorescence staining was performed as previously described (Wu et al., 2019b). Briefly, tumor and animal tissues were fixed with paraformaldehyde, dehydrated, and cleared with a gradient of alcohol solutions and xylene, respectively. Consecutively, tissues were embedded in paraffin. Paraffin-embedded tissues were sliced continuously (3.5°C) using a HistoCore AUTOCUT system (Leica), mounted, dewaxed, and rehydrated. For antigen retrieval, sections (3–4 μm) were pretreated in a microwave oven with citric acid buffer (pH 6.0) for 12 min and then cooled to 25°C in deionized water. After washing with PBS for 5 min, sections were incubated with 5% normal goat and donkey serum at 37°C for 1 h at 25°C and then incubated with the primary antibody (1:800, ab37647, Abcam) at 4°C overnight. After washing at least thrice with PBS, sections were incubated with the following secondary antibodies: Alexa 633-conjugated donkey anti-rat antibody (1:500, Invitrogen), Alexa 596-conjugated goat anti-rabbit (1:500, Invitrogen), and Alexa 488-conjugated goat anti-mouse antibody (1:250, Invitrogen) for 1 h at 25°C. Afterward, cells were extensively washed with PBS, and their nuclei were labeled using 4′,6-diamidino-2-phenylindole (DAPI). Free-floating sections with positive immunofluorescence staining were captured and analyzed using a laser scanning confocal microscope (Zeiss).

### Cell Counting Kit-8 Assay

Cell proliferation was measured using the Cell Counting Kit-8 (CCK-8) reagent (Solarbio), as previously described (Wang et al., 2021). Briefly, cells were cultured in 96-well plates with corresponding treatments. At 0, 24, 48, and 72 h, 10 µl CCK-8 reagent was added to each well. After 2 h of incubation at 37°C, the optical density at 450 nm was measured using a microplate reader.

### Statistical Analysis

Data were analyzed using the GraphPad Prism 8.0.2 software, SPM 12, and REST software. All quantitative data are shown as the mean ± SD of at least three independent experiments. Two-group comparisons were assessed using the Student’s *t*-test. Multigroup comparisons were analyzed using one-way ANOVA, followed by the Bonferroni *post-hoc* test on dependent experimental designs. Statistical significance was set at *p* < 0.05.

## Results

### Existence of TIGIT^+^ Treg Cells in Bladder Cancer Tissues

**FIGURE 1:**
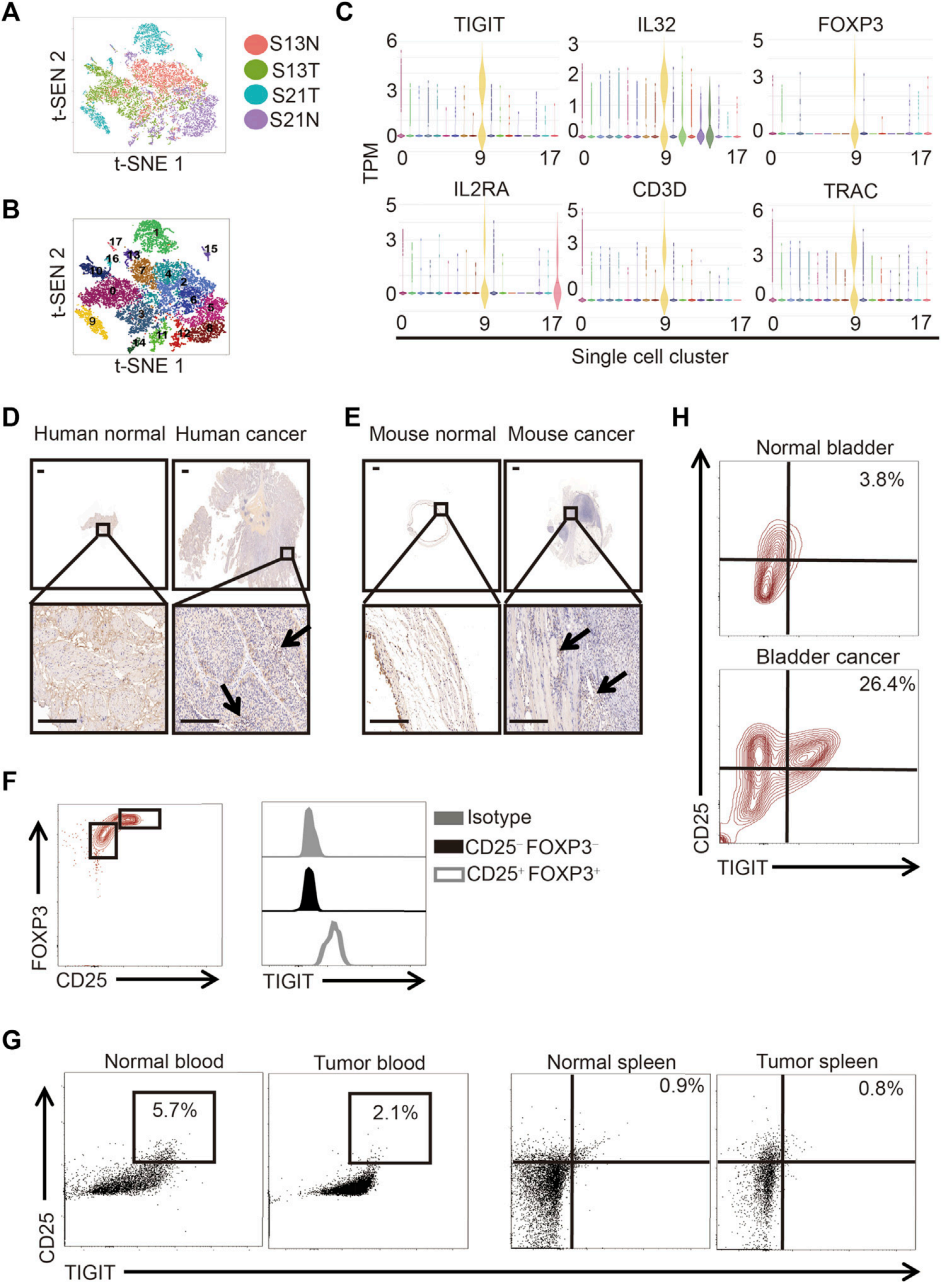
TIGIT^+^ Treg cells in bladder cancer tissues. **(A)** Schematic showing the single-nucleus RNA sequencing process. Samples of bladder tissues were surgically removed. Single cells were obtained from cancerous (n = 2) or paracancerous (n = 2) bladder tissues of patients and processed by plate-based scRNA-seq.**(B)** Two-dimensional t-distributed stochastic neighbor embedding (t-SNE) visualization from single-nucleus RNA sequencing showing the distribution of all nuclei. Eighteen major nuclear classes were identified. Each dot represents a single nucleus colored according to cluster assignment. Background is colored by major cell types (epithelial cells, T cells, B cells, endothelial cells, fibroblasts, stromal cells, umbrella cells, and smooth muscle cells). **(C)** Gene expression for each TIGIT + IL-32 + IL2RA + FOXP3+ CD3^+^ TRAC + single cell plotted as log2 counts per million. **(D,E)** Higher expressions of TIGIT in human **(D)** and murine **(E)** bladder cancer tissues. Immunohistochemistry images showing the expression of TIGIT in bladder tissues collected from different anatomical sites and groups of animals. Representative data from four independent experiments (n = 5). Scale bar = 1 mm. **(F)** Higher level of expression of TIGIT on the surface of Treg cells. Overlaid histogram plots showing the levels of expression of TIGIT in different CD4^+^ T-cell subsets. Data are representative of three independent experiments (n = 6). **(G)** TIGIT + Treg cells in the blood and spleen of mice with bladder cancer. The numbers in the dot plots represent the frequencies of TIGIT + Treg cells. Data are representative of three independent experiments (n = 8). **(H)** Higher frequencies of TIGIT + Treg cells in murine bladder cancer tissues. The numbers in the dot plots represent the frequencies of TIGIT + Treg cells. Data are representative of four independent experiments (n = 8).

To determine the cell composition of bladder cancer tissues, we compared the cells in clinical samples of bladder cancer tissues to those of paracancerous tissues using single-cell sequencing. Following the employment of PCA for the reduction of the dimensionality of single-cell gene expression profiles, in both bladder cancer and paracancerous tissues, we identified 18 clusters based on principal components. We used t-distributed stochastic neighbor embedding (t-SNE) to visualize the gene expression profiles (Figures 1A, B). In particular, we detected the presence of a subset of regulatory T (Treg) cells in bladder cancer tissues but not in paracancerous tissues (Figure 1B). Interestingly, we found that this subset of Treg cells not only highly expressed IL-2Rα (also called CD25) and forkhead box protein 3 (FOXP3) but also TIGIT and IL-32 (Figure 1C). To verify the protein expression of TIGIT in bladder cancer tissues, we performed immunohistochemistry (IHC) analysis of TIGIT in both bladder cancer and paracancerous tissues in patients and mice. We observed that in both human and mice, the expression of IL-32 was much higher in bladder cancer than in paracancerous tissues (Figures 1D, E). Furthermore, we examined the expression of TIGIT in Treg cells from murine bladder cancer tissues. As expected, TIGIT was highly expressed on the surface of Treg cells from bladder cancer tissues (Figure 1F). In addition, we detected higher frequencies of TIGIT^+^ Treg cells only in bladder cancer tissues but not in spleen tissues or peripheral blood cells (Figures 1G,H). Thus, TIGIT^+^ Treg cells were unambiguously enriched in bladder cancer tissues.

### Expression of IL-32 in Treg Cells in Bladder Cancer Tissues

**FIGURE 2:**
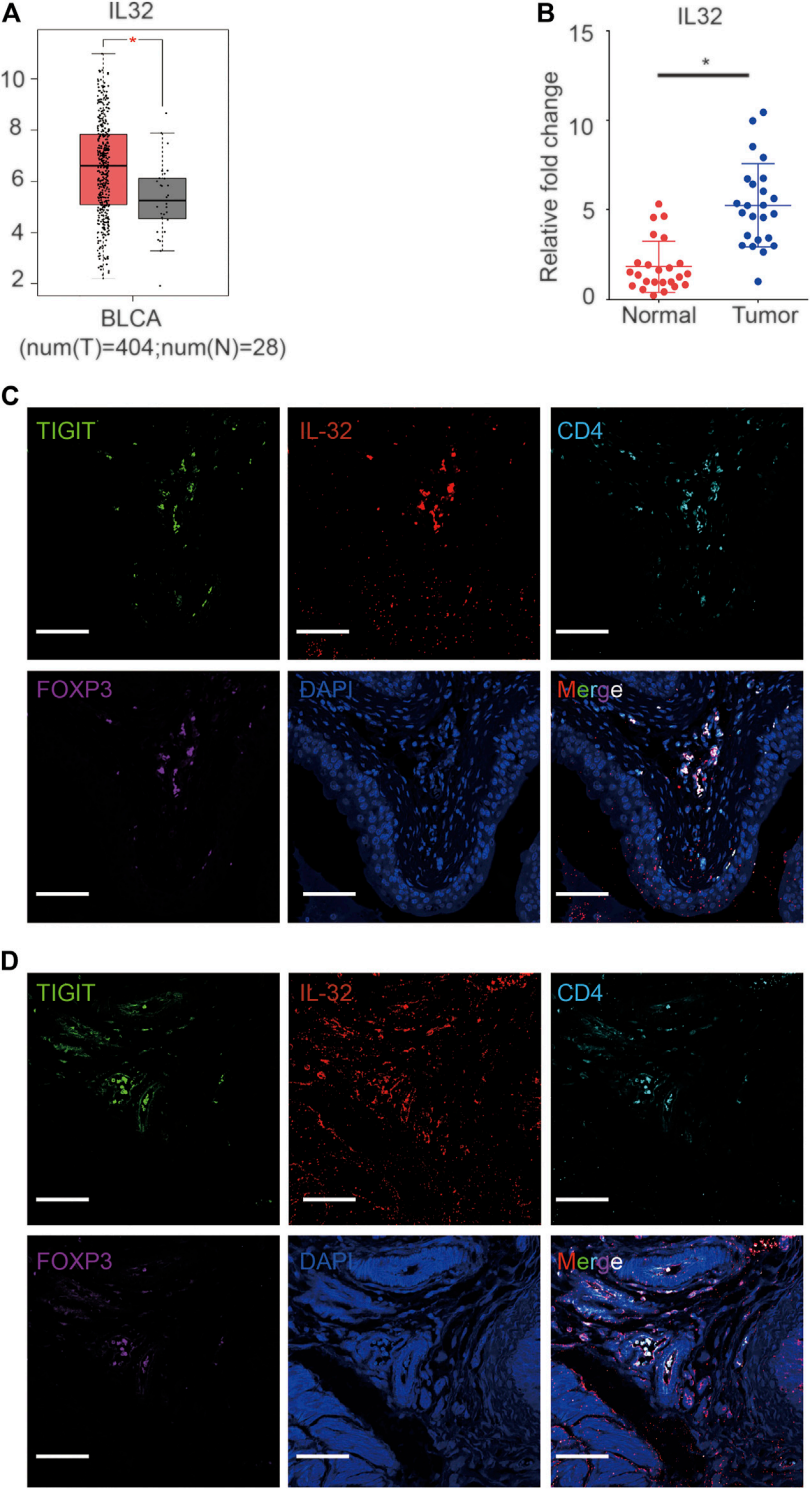
Expression of IL-32 in Treg cells in bladder cancer tissues. **(A)** Higher expression of IL-32 in the bladder cancer cohort in the TCGA and GTEx databases. Data are shown as the mean ± standard deviation (SD). *p < 0.05; *t*-test. **(B)** Higher relative expression of IL-32 in bladder cancer tissues. qRT-PCR analysis showing the relative expression of IL-32 in healthy and cancerous bladder tissues. Data are shown as the mean ± SD. *p < 0.05; *t*-test. **(C)** IL-32 colocalizes with Treg cells in human bladder cancer tissues. Representative immunofluorescence images showing cells stained with anti-FOXP3 (violet), IL-32 (red), CD4 (light blue), TIGIT (green), and DAPI (blue). Scale bar = 100 μm.**(D)** IL-32 colocalizes with Treg cells in murine bladder cancer tissues. Representative immunofluorescence images showing cells stained with anti-FOXP3 (violet), IL-32 (red), CD4 (light blue), TIGIT (green), and DAPI (blue). Scale bar = 100 μm.

Based on single-cell sequencing results, Tregs infiltrating bladder cancer tissues also expressed IL-32. To confirm the high expression of IL-32 in bladder cancer, we analyzed data from The Cancer Genome Atlas Program (TCGA) and The Genotype-Tissue Expression Project (GTEx) as previously reported (Tang et al., 2017). As expected, we noticed that the expression of IL-32 in the bladder cancer cohort was higher than that in the healthy cohort (Figure 2A). To validate the upregulation of IL-32 in bladder cancer tissues, we measured the expression of IL-32 using quantitative real-time PCR (qRT-PCR). We found that the expression of IL-32 was indeed upregulated in bladder cancer tissues to a certain extent (Figure 2B). However, whether IL-32 was expressed in Treg cells from bladder cancer tissues remains unknown. To verify the expression of IL-32 in Treg cells in bladder cancer tissues, we analyzed the abundances of IL-32, TIGIT, FOXP3, and CD25 in the TCGA and GTEx as previously reported (Tang et al., 2017). We accordingly detected that the expression of IL-32 was correlated with the abundance of TIGIT, FOXP3, and CD25 (Supplementary Figure S1). To validate the expression of IL-32 in Treg cells, we analyzed the colocalization of IL-32 and Treg cells using immunofluorescence. We found that IL-32 was colocalized with FOXP3 and TIGIT in clinical bladder cancer samples (Figure 2C). To establish an animal model to test the function of targeting TIGIT or IL-32 against bladder cancer, we inoculated C57B6/J mice with MBT-2 cells and analyzed the colocalization of IL-32 and Treg cells. As expected, we noticed that IL-32 was also colocalized with FOXP3 and TIGIT in murine bladder cancer tissues (Figure 2D). Collectively, our data suggested that IL-32 is highly expressed in Treg cells in bladder cancer tissues.

### IL-32 Promoted the Metastasis of Bladder Cancer

**FIGURE 3:**
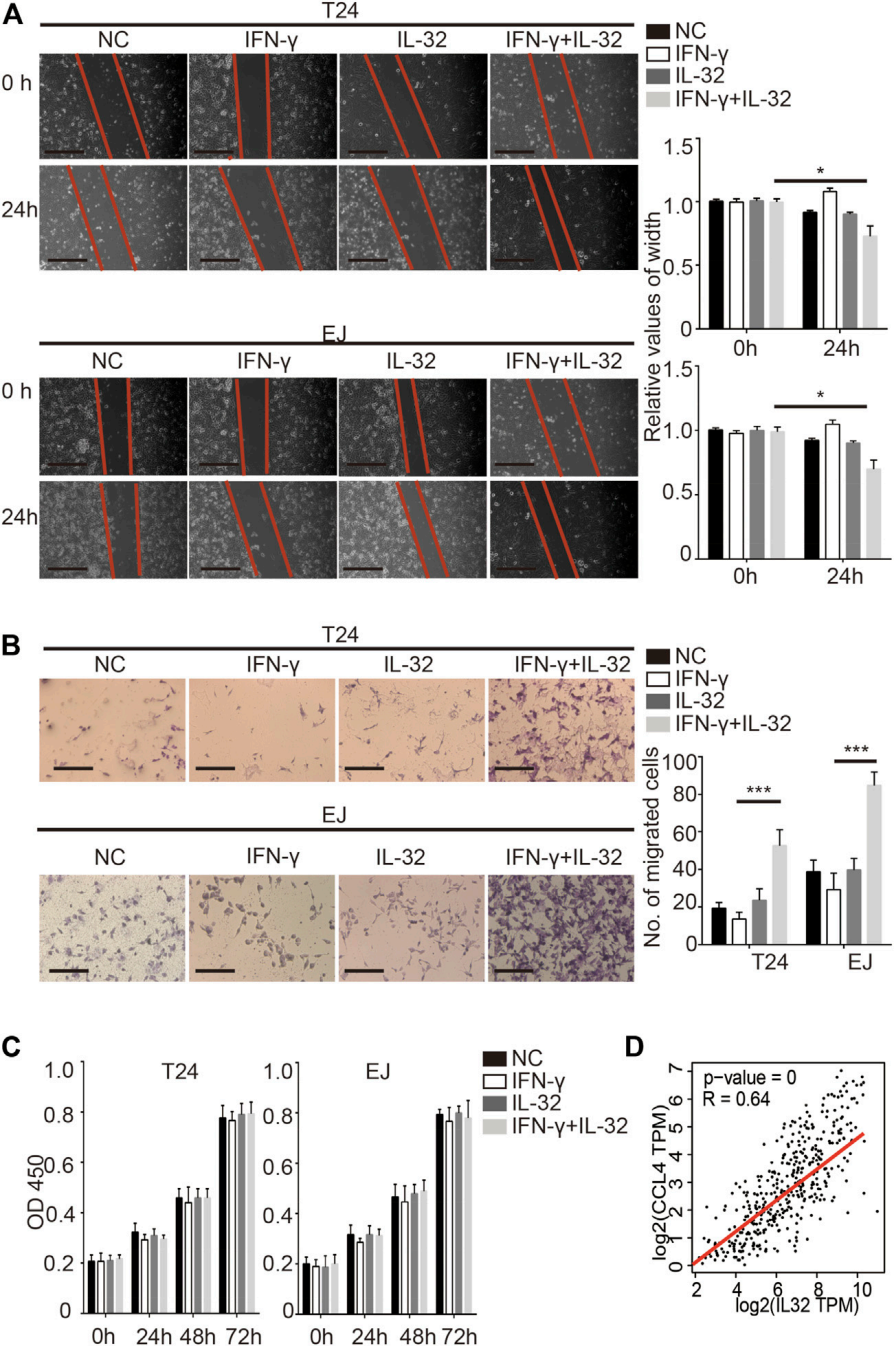
IL-32 promotes the metastasis of bladder cancer. **(A)** IL-32 enhances the migration ability of bladder cancer cells. Representative data of 3 independent experiments from wound healing migration assays performed with the indicated bladder cancer cells. Scale bar = 100 μm **(left panel)**. The relative values of width are shown as the mean ± SD. *p < 0.05; one-way ANOVA **(right panel)**. **(B)** IL-32 increases the invasion ability of bladder cancer cells. Representative data of three independent experiments from crystal violet staining showing the invasive capacities of bladder cancer cells. Scale bar = 100 μm **(left panel)**. The number of migrated cells is shown as the mean ± SD. ***p < 0.001; one-way ANOVA **(right panel)**. **(C)** IL-32 does not disrupt the proliferation of bladder cancer cells. Cell viability was measured using the CCK8 assay. Data are shown as the mean ± SD. **(D)** The association between IL-32 and CCL4. The relationship between IL-32 and CCL4 in the TCGA and GTEx databases is shown. Spearman’s rank correlation coefficient is shown.

As previously reported, IL-32 has multiple potential functions (Hong et al., 2017). To explore the specific function of IL-32 in bladder cancer, we incubated bladder cancer cells with a minimal concentration of IL-32 to mimic the effect of the secretion of IL-32 from Treg cells on bladder cancer cells. Similar to the effect of IL-32 on colorectal cancer, we found that IL-32 enhanced the migration of T24 and EJ cells (Figure 3A) (Yang et al., 2015). Moreover, IL-32 increased the invasion of bladder cancer cells (Figure 3B). However, we observed that IL-32 did not mediate the proliferation of bladder cancer cells (Figure 3C). To explore the mechanism of the IL-32-mediated invasion and migration of bladder cancer cells, we analyzed the relationship between the abundance of IL-32 and the expression of molecules that mediate the invasion and migration of bladder cancer cells in the TCGA and GTEx databases (Tang et al., 2017). We found that the expression of IL-32 was associated with the abundance of the C–C motif chemokine ligand 4 (CCL4) (Figure 3D). Hence, IL-32 promoted the metastasis of bladder cancer.

### Targeting TIGIT Suppressed Bladder Cancer

**FIGURE 4:**
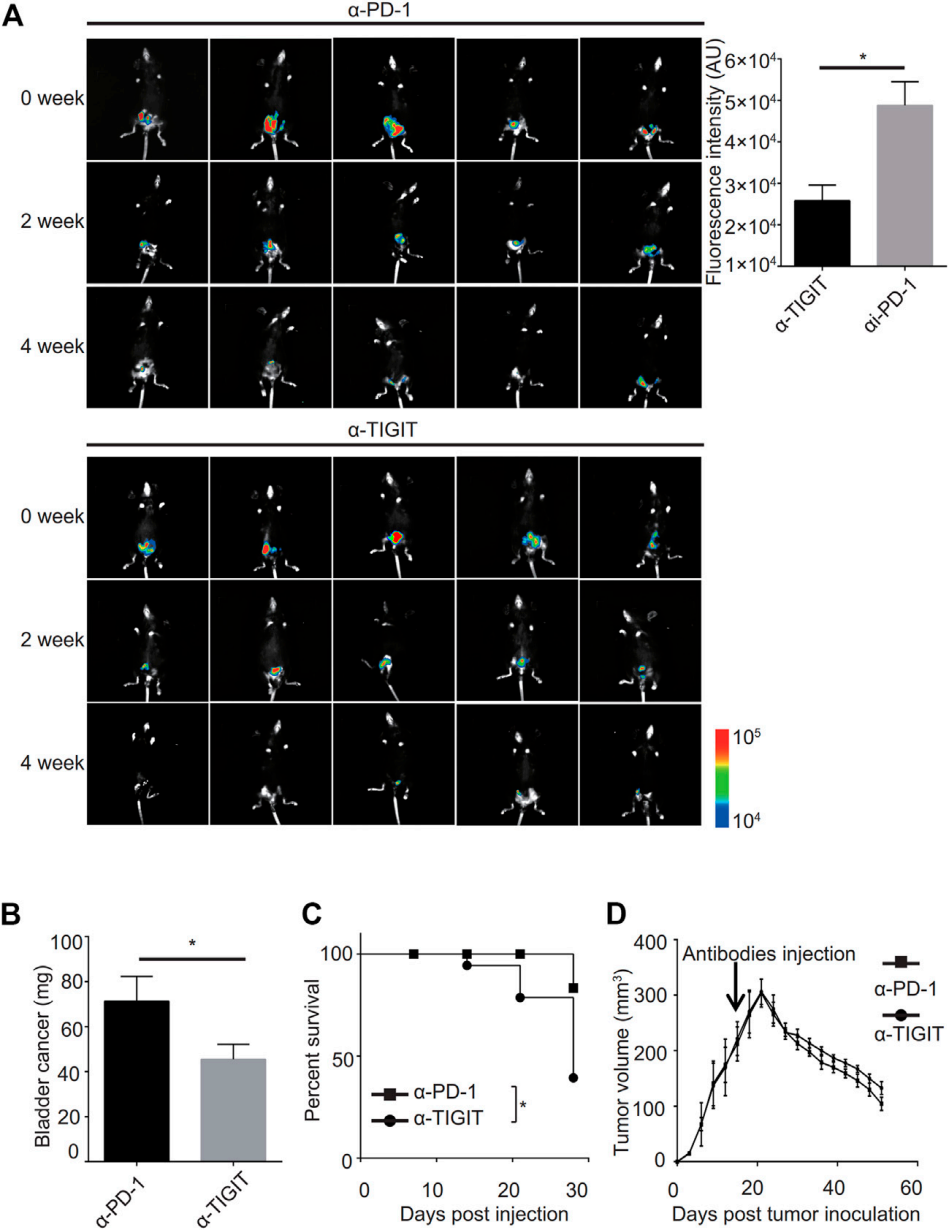
Targeting TIGIT suppressed bladder cancer. **(A**, **B)** α-TIGIT suppresses bladder cancer. *In vivo* bioluminescent imaging of C57B6/J mice transplanted with MBT2-luciferase cells **(left panel)**. Bioluminescence intensities are shown as the mean ± SD. *p < 0.05; *t*-test. Data are representative of three independent experiments (n = 8) **(right panel)**. **(C)** α-TIGIT suppresses the growth of bladder tissues. Bladder weight of mice are shown as the mean ± SD. *p < 0.05; *t*-test. Data are representative of three independent experiments (n = 8). **(D)** Tumor-bearing mice administered α-TIGIT show a better prognosis. The relative survival rates of mice in different groups were recorded. Data are representative of four independent experiments (n = 6). *p < 0.05; log-rank (Mantel-Cox) test.

Given the good performance of the monoclonal antibody of TIGIT (α-TIGIT) against some carcinomas, we explored the antitumor capacity of TIGIT antibodies against bladder cancer. To ensure the specificity of α-TIGIT and minimize any side effects, we analyzed the distribution of TIGIT in the whole body of patients. Using the Protein Atlas database, we detected that the expression of TIGIT was higher in the urinary tract than in other tissues, except for lymphatic tissues (Supplementary Figure S2). Thus, we aimed to establish a bladder cancer orthotropic model and then compare the antitumor capacity of α-PD-1 and α-TIGIT. To visually compare the long-term efficacy of these antibodies, we established a murine model transfected with luciferase-expressing MBT-2 cells. Then, 18 days after inoculation, we intravenously administered mice with α-PD-1 or α-TIGIT (Figure 4A). We found that the tumor size in mice administered α-TIGIT was suppressed to a greater extent compared with that in mice administered α-PD-1 (Figure 4A). In addition, we observed that compared with mice injected with α-PD-1, the weights of bladders in mice administered α-TIGIT were greatly reduced (Figure 4B). Moreover, the survival rates of mice administered α-TIGIT were also higher than those of mice administered α-PD-1 (Figure 4C). Surprisingly, we did not observe any significant difference between mice administered a-TIGIT or a-PD-1 in the subcutaneous tumor model (Figure 4D). Our results clearly demonstrated that targeting TIGIT suppressed bladder cancer.

### Targeting TIGIT Promoted the Capability of Antitumor of T Cells

**FIGURE 5:**
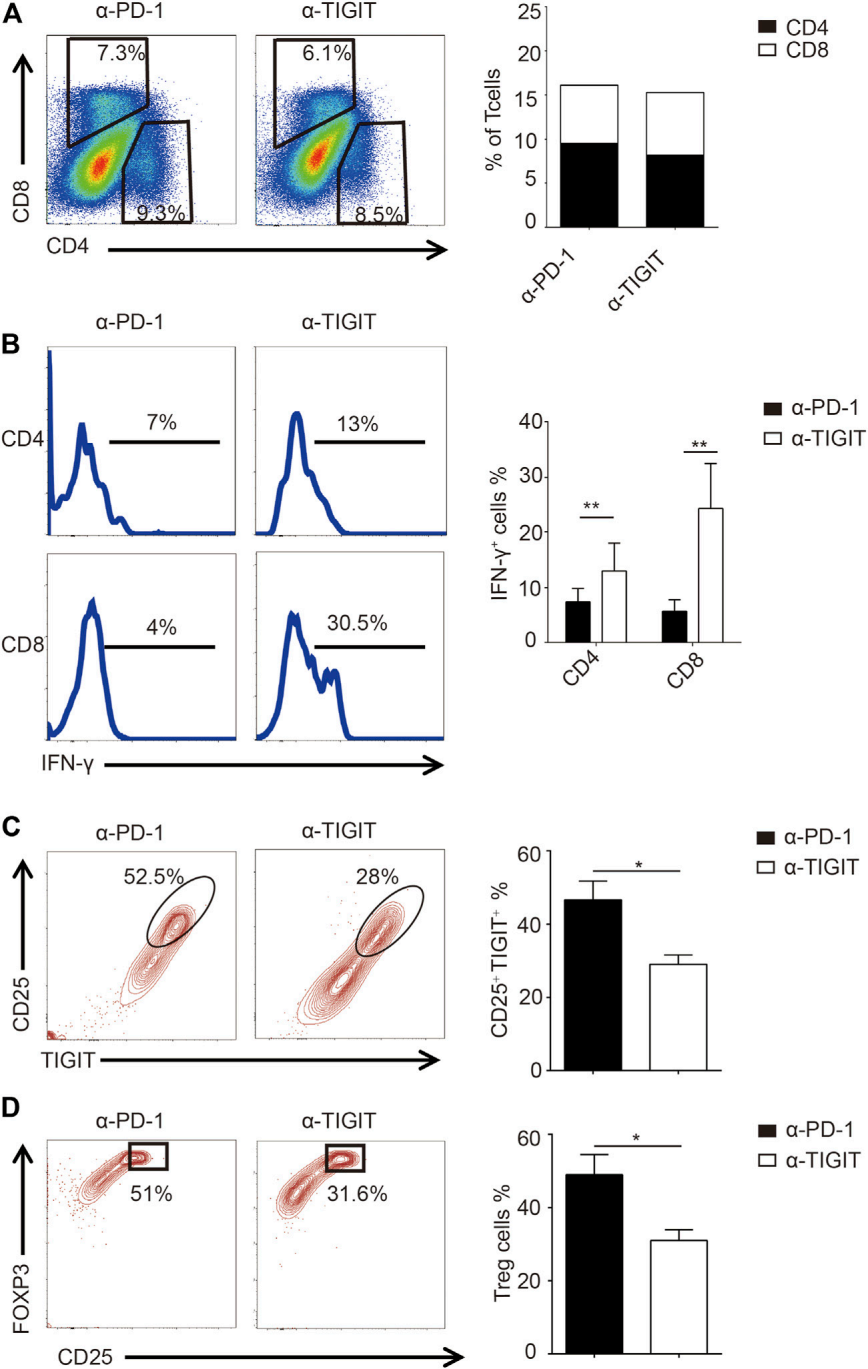
Targeting TIGIT promoted the antitumor capability of T cells. **(A)** Administration of α-TIGIT does not affect the ratios of CD4^+^ and CD8^+^ T-cells in bladder cancer tissues. Dot plots represent the frequencies of CD4^+^ and CD8^+^ T cells in murine bladder cancer tissues from three independent experiments (n = 6) **(left panel)**. The ratios of CD4^+^ and CD8^+^ T cells are shown as the mean **(right panel)**. **(B)** Administration of α-TIGIT upregulates the secretion of IFN-γ in T cells. Numbers in histograms show the representative ratios of IFN-γ+ T cells from three independent experiments (n = 6) **(left panel)**. The ratios of IFN-γ+ cells are shown as the mean ± SD. **p < 0.01; one-way ANOVA **(right panel)**. **(C)** Administration of α-TIGIT suppresses the expression of TIGIT in Treg cells. Dot plots represent the ratios of TIGIT + CD25 ^+^ subsets in CD4^+^ T cells from three independent experiments (n = 6) **(left panel)**. The ratios of TIGIT + CD25 ^+^ cells are shown as the mean ± SD. *p < 0.05; *t*-test **(right panel)**. **(D)** Administration of α-TIGIT suppresses the generation of Treg cells in mice with bladder cancer. Dot plots represent the ratios of CD25 ^+^ FOXP3+ subsets in CD4^+^ T cells from three independent experiments (n = 6) **(left panel)**. The ratios of CD25 ^+^ FOXP3+ cells are shown as the mean ± SD. *p < 0.05; *t*-test **(right panel)**.

Given the efficiency of α-TIGIT against bladder cancer, we investigated the mechanisms underlying the antitumor effects of α-TIGIT. As TIGIT is known to be functionally related to the immune system, we compared the immune activities of mice administered with α-PD-1 or α-TIGIT. We noticed that the ratios of CD4^+^ and CD8^+^ T cells were not disrupted by the administration of α-PD-1 or α-TIGIT (Figure 5A). However, we found that IFN-γ was upregulated in CD4^+^ and CD8^+^ T cells of mice administered α-TIGIT compared with that in mice administered α-PD-1 (Figure 5B). As with the enhancement of the tumor-killing capacities of immune cells, the changes in immunosuppressive cells caused by the tumor microenvironment, especially the changes in Treg cells, remain unknown. To explore the regulation of α-TIGIT in Treg cells, we analyzed the expression of CD25, TIGIT, and FOXP3 in murine bladder cancer tissues. Flow cytometric analysis showed that the ratio of TIGIT^+^ CD25^+^ CD4^+^ T cells was downregulated in those tissues following the administration of α-TIGIT (Figure 5C). Similarly, we noticed that the ratio of Treg cells (CD25^+^ FOXP3^+^) in bladder cancer tissues was also downregulated after the administration of α-TIGIT (Figure 5D). Collectively, these data indicated that targeting TIGIT with α-TIGIT upregulated the antitumor capability of T cells, whereas it downregulated the induction of immunosuppression.

### Targeting TIGIT Inhibited the Metastasis of Bladder Cancer Through Suppressing IL-32

**FIGURE 6:**
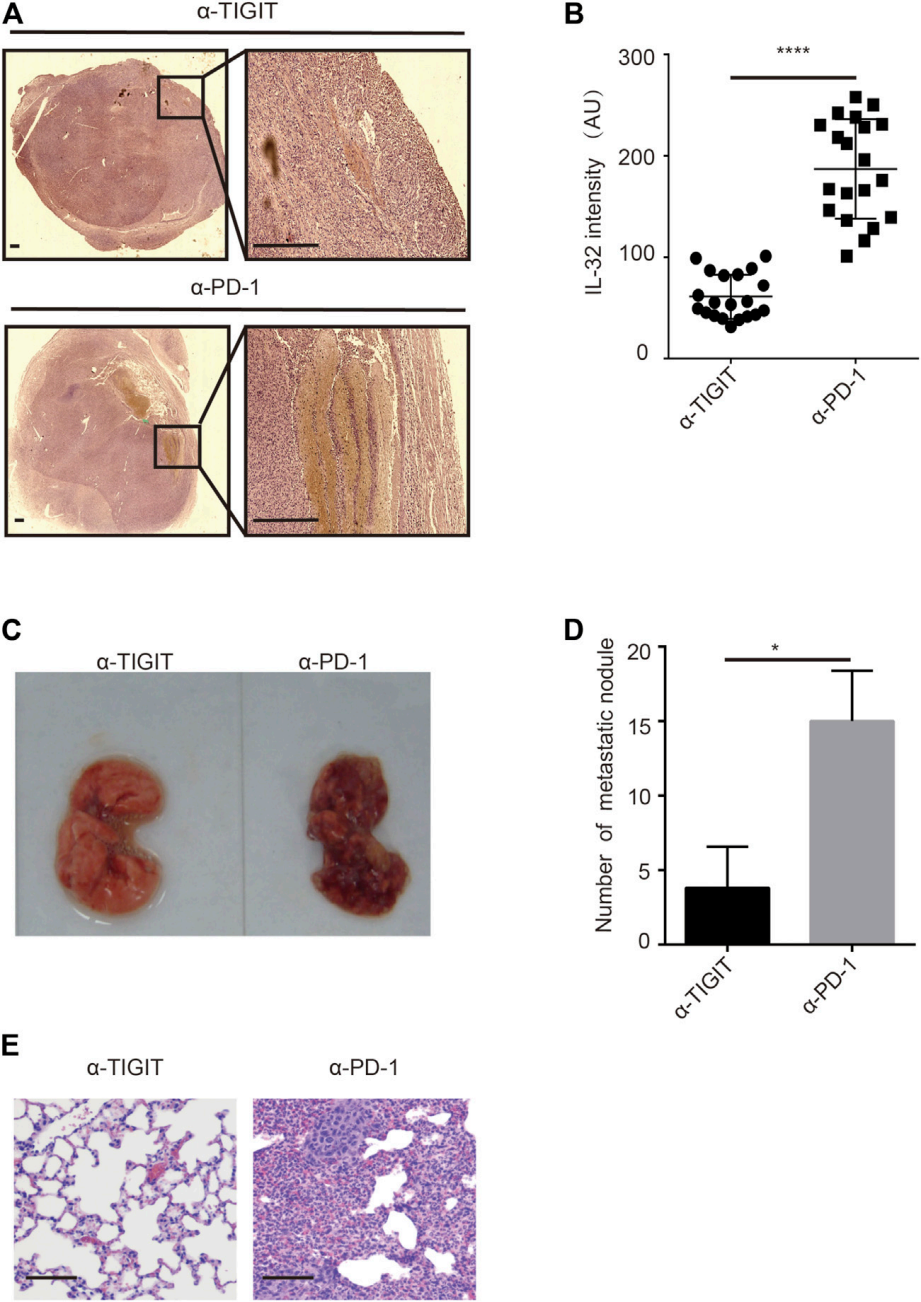
Targeting TIGIT inhibits the metastasis of bladder cancer through suppressing IL-32. **(A**,**B)** Targeting TIGIT suppresses the expression of IL-32. Immunohistochemistry images showing the expression of IL-32 in bladder tissues collected from different groups of animals. Scale bar = 1 mm **(A)**. The levels of expression of IL-32 are shown as the mean ± SD. ****p < 0.0001; *t*-test **(B)**. Data are representative of four independent experiments (n = 7). **(C−E)** Targeting TIGIT inhibits the metastasis of bladder cancer. Metastatic nodules in lungs of mice inoculated with bladder cancer cell lines **(C)**. The number of metastatic nodules is shown as the mean ± SD. *p < 0.05; *t*-test **(D)**. Images showing HE-stained bladder tissues collected from different groups of animals. Scale bar = 100 μm **(E)**. Data are representative of four independent experiments (n = 7).

Given the high abundance of IL-32 in Treg cells in bladder cancer tissues, we investigated whether targeting TIGIT with α-TIGIT suppresses the secretion of IL-32. As expected, we found that administration of α-TIGIT antibody suppressed the expression of IL-32 in bladder cancer tissues 3 days post-injection (Figures 6A,B). Because IL-32 mediates the metastasis and invasion of bladder cancer cells, targeting TIGIT might inhibit the metastasis of bladder cancer by suppressing the expression of IL-32. To verify this hypothesis, we intravenously injected MBT-2 cells into mice to establish a bladder cancer metastasis mouse model. After 3 days, mice were intravenously injected with α-TIGIT or α-PD-1. We accordingly detected that administration of α-TIGIT significantly reduced the number of metastatic nodules (Figures 6C–E). Thus, our data indicated that targeting TIGIT inhibited the metastasis of bladder cancer cells by suppressing the expression of IL-32.

## Discussion

In this study, we identified a specific subset of Treg cells in human bladder cancer tissues that highly expressed TIGIT and IL-32. Furthermore, IL-32 from Treg cells promoted the metastasis of bladder cancer cells, while Treg cells mediated immunosuppression. Conversely, targeting TIGIT with anti-TIGIT antibodies enhanced the antitumor immune capacity of the host against bladder cancer. Importantly, targeting TIGIT with anti-TIGIT antibodies not only enhanced the antitumor activities of T cells but also suppressed the abundance of IL-32, in turn inhibiting the metastasis of bladder cancer cells. Thus, we have demonstrated a novel function of Treg cells in bladder cancer tissues. Hence, as an immune checkpoint, TIGIT not only enhances the antitumor immune response but also inhibits the metastasis of bladder cancer cells by suppressing the expression of IL-32.

The main function of regulatory T cells (Tregs) is to regulate immune responses, especially immunosuppression; however, functions other than immunological functions have rarely been reported (Togashi et al., 2019). In our study, we identified the accessory nonimmune function of Treg cells, that is, secreting IL-32 to promote the metastasis of bladder cancer cells. Our study showed that as immunosuppressive cells, Treg cells mediate tumor metastasis in a different way from tumor-related macrophages (Lin et al., 2019). Instead of carrying tumor cell metastasis, they secrete cytokines that mediate tumor metastasis. Meanwhile, their immunosuppressive function, that is, their canonical function, was preserved in bladder cancer tissues.

Antitumor immunotherapy is one of the most effective tumor treatments after chemotherapy and radiotherapy, but its effective rate is almost impossible to exceed 50% (Kruger et al., 2019). This is because current therapeutic targets are not suitable for all patients. In the era of precision medicine, new immunotherapy targets can be identified through sequencing technology (Zhang et al., 2021). In this study, using single-cell sequencing technology, we found that Treg cells in bladder cancer tissues highly expressed TIGIT, demonstrating it as a more suitable therapeutic target. Fortunately, targeting TIGIT had a dual effect, not only upregulating the immune response against bladder cancer but also inhibiting its metastasis. Thus, our study confirmed that this is a new way to develop and verify new immunotherapeutic targets. In particular, the CTLA-4 antibody is usually adapted to depleted Treg cells. However, administration of CTLA-4 antibodies did not downregulate the ratio of Treg cells in bladder cancer (Rouanne et al., 2018). Therefore, application of TIGIT antibodies might provide an effective option for the depletion of Treg cells in bladder cancer tissues to enhance antitumor activities. Surprisingly, in the subcutaneous tumor model, administration of a-TIGIT did not achieve better effects than injection of a-PD-1; we speculated that this was because only the bladder tumor microenvironment exhibited a higher expression of TIGIT. More importantly, we used TIGIT monoclonal antibodies as a single agent against bladder cancer and did not explore whether their combination with other drugs could achieve a better curative effect. As such, the efficacy of TIGIT in clinical practice remains inconclusive, and further research is needed.

The origin of IL-32 has been reported in different cells from distinct tumors. In contrast to breast cancer and small cell lung cancer, we have identified that IL-32 is derived from Treg cells in bladder cancer tissues (Sorrentino and Di Carlo, 2009). However, although the expression of IL-32 was positively correlated with Treg cell markers, the relationship between the expression of IL-32 and the prognosis of patients with bladder cancer in clinical practice remains unknown. Furthermore, the function of IL-32 in tumors is complex and controversial. In this study, we verified the positive effect of IL-32 for the invasion and migration of bladder cancer cells *in vitro* and identified the correlation between IL-32 and CCL4 (as a chemoattractant induced by inflammation, also known as macrophage inflammatory protein-1β) according to the TCGA and GTEx databases, but its function *in vivo* remains unknown. IL-32 is known to be an inflammation factor (Hong et al., 2017). However, the specific pathway by which IL-32 mediates the migration and invasion of tumor cells remains unknown. Meanwhile, it also remains unknown whether IL-32 first induces inflammation and then causes tumor metastasis in bladder cancer, especially by inducing the generation of cancer-associated fibroblasts through inflammation. In addition, although the TIGIT antibodies used in this study achieved a good effect of inhibiting the abundance of IL-32, the direct development of IL-32 monoclonal antibodies for the inhibition of IL-32 is a very attractive therapeutic option. Of note, regardless of breast cancer, colorectal cancer, and bladder cancer, the expression of IL-32 has been identified in several tumor tissues (Diakowska and Krzystek-Korpacka, 2020). However, IL-32 exhibits flowing characteristics and remains unclear whether it can be detected in blood or urine to be used as a diagnostic marker.

Overall, our study revealed the existence of TIGIT^+^ IL-32^+^ Treg cells in bladder cancer tissues. These cells not only suppressed antitumor immunoresponses but also promoted tumor metastasis. Surprisingly, targeting TIGIT not only relieved immunosuppression and enhanced immunity but also inhibited tumor metastasis, providing a double therapeutic benefit. Thus, our study provided an alternative option for bladder cancer therapy.

## Data Availability

The datasets presented in this study can be found in online repositories. The names of the repository/repositories and accession number(s) can be found below: https://www.ncbi.nlm.nih.gov/geo, GSE186520.
