# Supplementary material for: Targeting TIGIT Inhibits Bladder Cancer Metastasis Through Suppressing IL-32
Source: Front Pharmacol. 2022 Jan 5;12:801493. doi: 10.3389/fphar.2021.801493 (PMC8766971; doi:10.3389/fphar.2021.801493)
Supplement: Supplementary file 1 [file DataSheet2.DOCX]

All data submitted as following link：

<https://www.jianguoyun.com/p/DR9mtsIQm7P7CRjE4ZcE>

All sequencing data as following：

GE0 number：GSE186520 token：kzmdaiawjjynbup

link：https://www.ncbi.nlm.nih.gov/geo/info/linking.html
