# Supplementary material for: Targeting TIGIT Inhibits Bladder Cancer Metastasis Through Suppressing IL-32
Source: Front Pharmacol. 2022 Jan 5;12:801493. doi: 10.3389/fphar.2021.801493 (PMC8766971; doi:10.3389/fphar.2021.801493)
Supplement: Supplementary file 2 [file DataSheet1.DOCX]

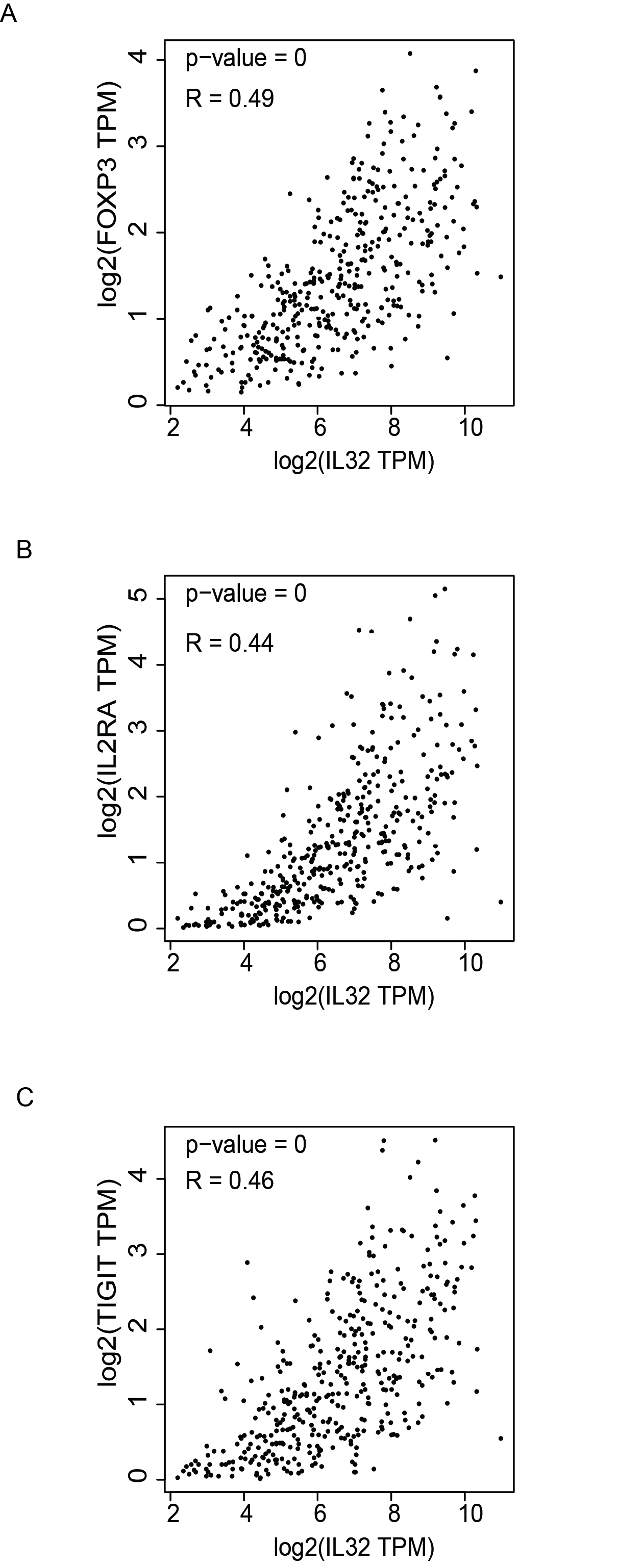


**Figure S1. Associations between IL32 and TIGIT, IL2RA as well as FOXP3 molecules in bladder cancer.**

The correlation between IL32 and FOXP3 molecule (A), IL2RA molecule (B), TIGIT molecule(C). Spearman’s rank correlation coefficients are shown.


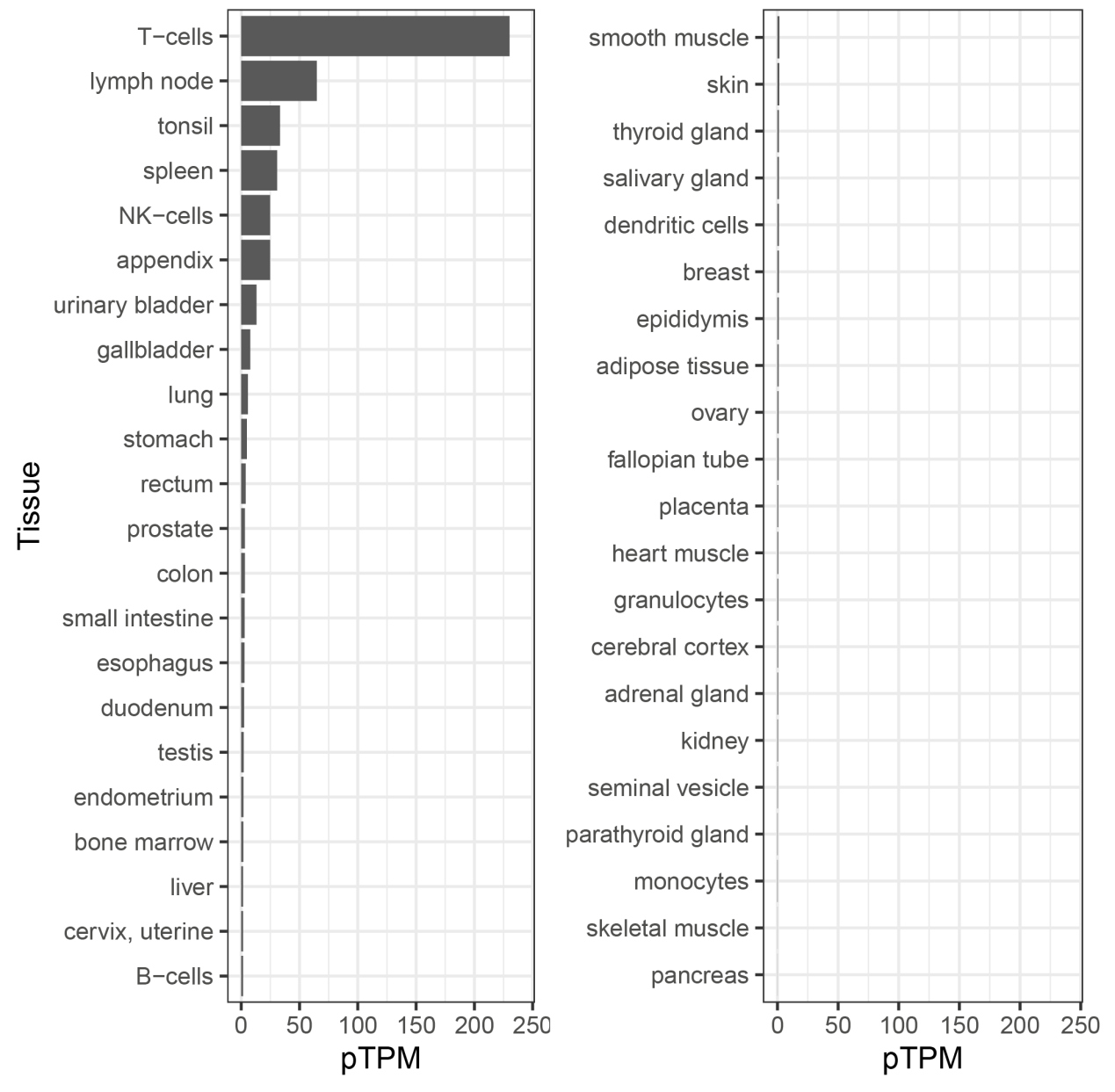


**Figure S2. The distribution of TIGIT molecule.**

The relative expressions of TIGIT molecule in different organs are shown as means.

**Supplemental Table Ⅰ. Clinical features of the 24 urothelial bladder carcinoma cases.**

| Sample | Sex^#^ | Age | T | N | M | TNM | NMIBC/  MIBC | Grade^*^ | Primary/  Relapsed |
| --- | --- | --- | --- | --- | --- | --- | --- | --- | --- |
| BL01 | F | 48 | 3 | 2 | 1 | T3N2M1 | MIBC | High | Primary |
| BL02 | M | 62 | 1 | 0 | 0 | T1N0M0 | NMIBC | Low | Primary |
| BL03 | M | 64 | 1 | 0 | 0 | T1N0M0 | NMIBC | Low | Primary |
| BL04 | M | 61 | 2 | 0 | 0 | T2N0M0 | MIBC | High | Primary |
| BL05 | M | 73 | 3 | 2 | 0 | T3N2M0 | MIBC | High | Primary |
| BL06 | M | 62 | 2 | 2 | 1 | T2N2M1 | MIBC | High | Primary |
| BL07 | M | 47 | 2 | 0 | 0 | T2N0M0 | MIBC | High | Primary |
| BL08 | M | 55 | 2 | 0 | 0 | T2N0M0 | MIBC | High | Primary |
| BL09 | F | 67 | 2 | 0 | 0 | T2N0M0 | NMIBC | High | Primary |
| BL10 | M | 71 | 2 | 0 | 0 | T2N0M0 | MIBC | High | Primary |
| BL11 | M | 74 | 3 | 1 | 0 | T3N1M0 | MIBC | High | Relapsed |
| BL12 | M | 61 | 1 | 0 | 0 | T1N0M0 | NMIBC | Low | Primary |
| BL13 | F | 63 | 1 | 0 | 0 | T1N0M0 | NMIBC | Low | Primary |
| BL14 | M | 56 | 1 | 0 | 0 | T1N0M0 | MIBC | Low | Primary |
| BL15 | M | 72 | 2 | 0 | 0 | T2N0M0 | MIBC | High | Primary |
| BL16 | M | 61 | 2 | 0 | 0 | T2N0M0 | MIBC | High | Primary |
| BL17 | M | 53 | 4 | 0 | 0 | T4N0M0 | MIBC | High | Primary |
| BL18 | M | 65 | 4 | 0 | 0 | T4N0M0 | MIBC | High | Relapsed |
| BL19 | M | 78 | 2 | 0 | 0 | T2N0M0 | MIBC | High | Primary |
| BL20 | M | 69 | 2 | 0 | 0 | T2N0M0 | MIBC | High | Primary |
| BL21 | M | 62 | 4 | 0 | 0 | T4N0M0 | MIBC | High | Relapsed |
| BL22 | M | 58 | 2 | 0 | 0 | T2N0M0 | MIBC | High | Primary |
| BL23 | F | 64 | 2 | 0 | 0 | T2N0M0 | NMIBC | High | Primary |
| BL24 | M | 76 | 2 | 0 | 0 | T2N0M0 | MIBC | High | Primary |

^#^M, Male; F, Female.

^*^PUNLMP, papillary urothelial malignancy of low malignant potential; Low, low-grade papillary urothelial carcinoma; High, high-grade papillary urothelial carcinoma (WHO/ISUP 2004 classification).
